# Supplementary material for: Potential of Epidermal Growth Factor-like Peptide from the Sea Cucumber Stichopus horrens to Increase the Growth of Human Cells: In Silico Molecular Docking Approach
Source: Mar Drugs. 2022 Sep 23;20(10):596. doi: 10.3390/md20100596 (PMC9605497; doi:10.3390/md20100596)

Supplementary Material Figure S3

RMSD of Sh-EGFl-1 model from the starting structure as a function of simulation time in nanoseconds

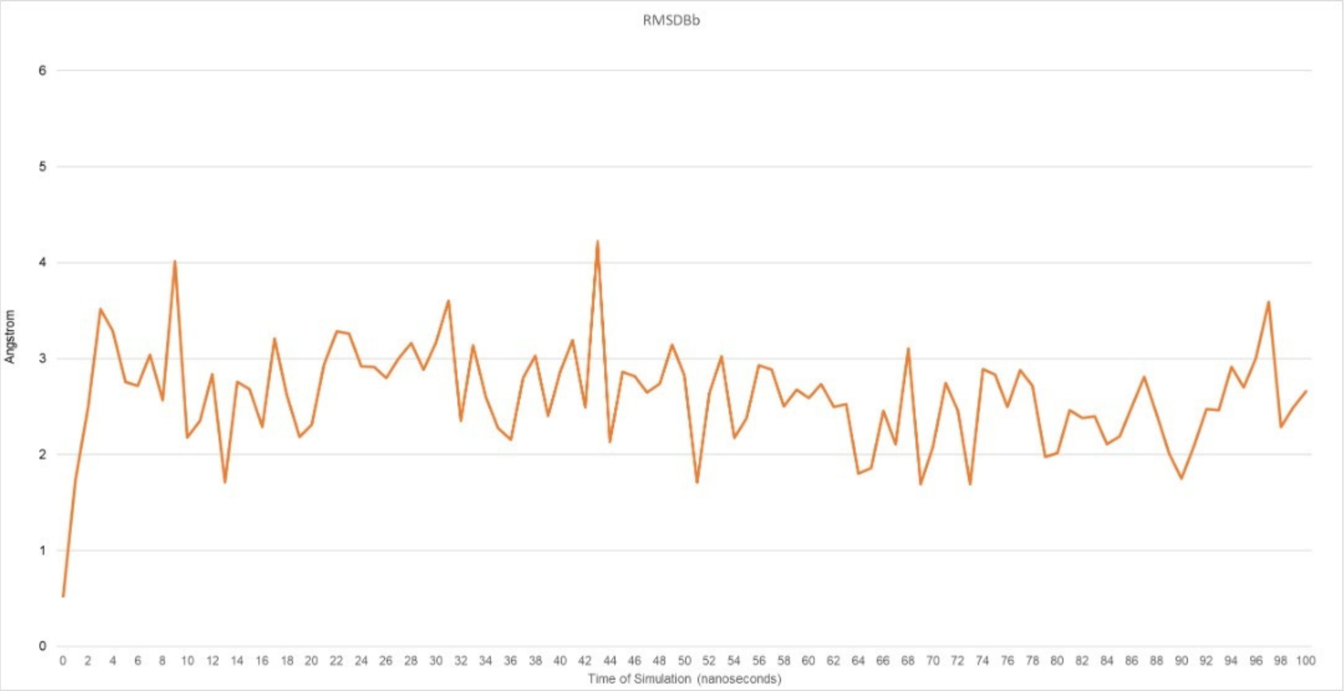

Supplement: Supplementary file 1 [file marinedrugs-20-00596-s001.zip › Supplementary Figure S3 - RMSD of Sh-EGFl-1 model.pdf]
