# Supplementary material for: Potential of Epidermal Growth Factor-like Peptide from the Sea Cucumber Stichopus horrens to Increase the Growth of Human Cells: In Silico Molecular Docking Approach
Source: Mar Drugs. 2022 Sep 23;20(10):596. doi: 10.3390/md20100596 (PMC9605497; doi:10.3390/md20100596)
Supplement: Supplementary file 1 [file marinedrugs-20-00596-s001.zip › Supplementary Figure S6 - Cell morphology observations.pdf]

Supplementary Material Figure S6

Cell Morphology Observations

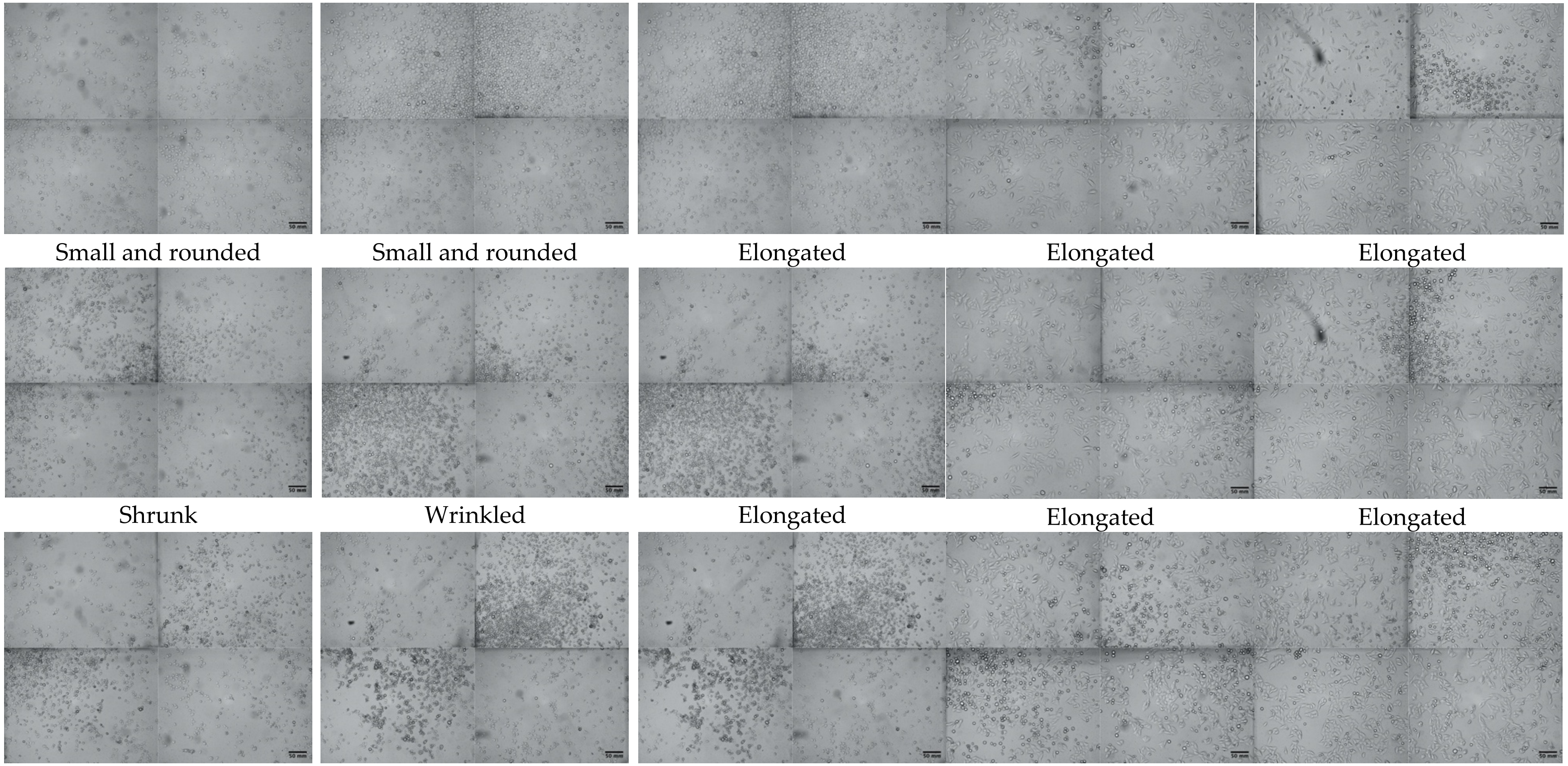

Time (hour)	Negative control	10 nM rhEGF	50 nM rhEGF	10 nM Sh-hEGFl-1	50 nM Sh-hEGFl-1
0	Small and rounded	Small and rounded	Elongated	Elongated	Elongated
24	Shrunk	Wrinkled	Elongated	Elongated	Elongated
48	Shrunk	Wrinkled	Elongated	Elongated but minority started to be rounded	Elongated but minority started to be rounded

72	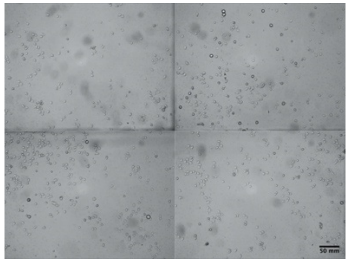	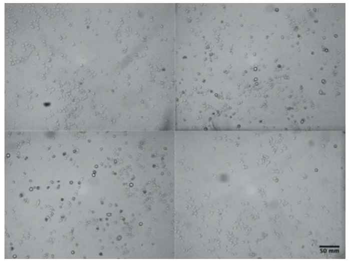	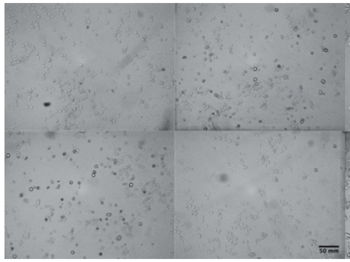	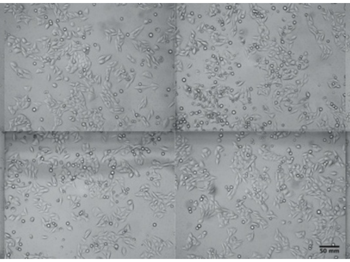	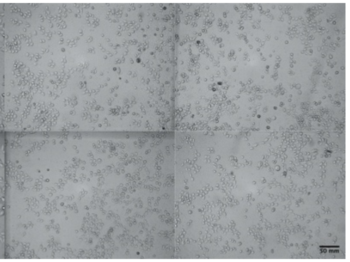
	Wrinkled	Small and rounded	Small and rounded	Elongated and not so much different on the number of rounded cells	Small and rounded
96	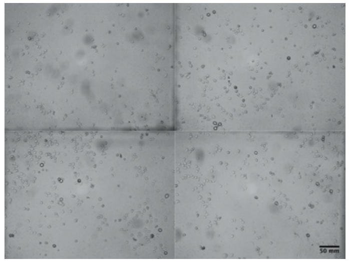	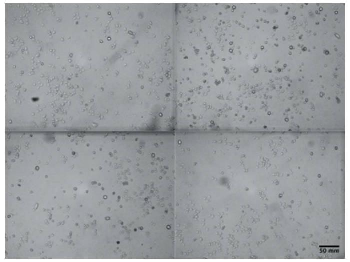	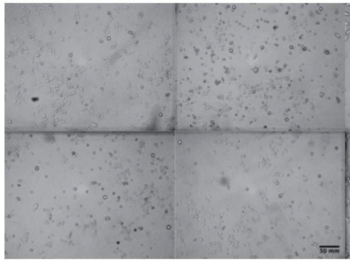	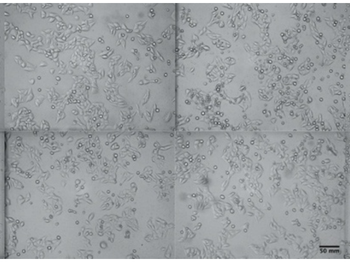	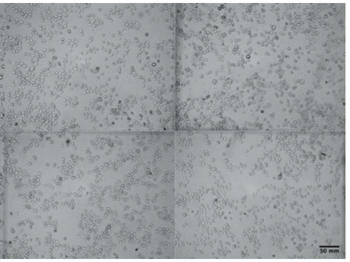
	Wrinkled	Wrinkled	Small and rounded	Elongated, minority of cells are wrinkled	Wrinkled

All images are taken using 10x magnification
