# Supplementary material for: Potential of Epidermal Growth Factor-like Peptide from the Sea Cucumber Stichopus horrens to Increase the Growth of Human Cells: In Silico Molecular Docking Approach
Source: Mar Drugs. 2022 Sep 23;20(10):596. doi: 10.3390/md20100596 (PMC9605497; doi:10.3390/md20100596)
Supplement: Supplementary file 1 [file marinedrugs-20-00596-s001.zip › Supplementary Materials S1 - S. horrens protein modeling.pdf]

Supplementary Material S1

S. horrens Protein Modeling

A) Prediction of *S. horrens* protein sequence

Sequence information of Contig 498513 derived from transcriptome data of *Stichopus horren* are as follow;

>Ct498513 Average coverage: 17.86
TGATGGGTGGGTAAGTGATCTCAATCTCACTGAGCTATTTTTGATCTGGCCTTTCTTAGA
GTAACCAAAGACAATGGAGTGCCTGAATGACATGGTTCAACATACTTGATTCACAATGA
GTGCCAGTAAACCCAGGCTGACAGACACAGTTATATCCATCGATCTCATCTTGGCAGAT
ACCCTGGTTCTCACAGGGGCTGCTGAAGCATTCGTTGATGTTGGTGCTGCAGGTAGAG
TCGGTCCAACCGGCAGCGCAGATGCAGACAAACCCGTTGACCTGATCAATGCAGGTGC
TGTTATGCAGGCAAGGAGTTTGGGAACACTCCGAAATTGCTGCAGAAAGAAATGTTTGT
AATTGTAGACATTTTTCTTGTTTGTAATTATTGAATGGAAATTTGCACAAAAATGATATGA
AATGACTCAAATACATAATCACTTTCATAACAATCAGTCACTATCAATAAAATATTTTGAT
ACATTTGCCACTGTGGTTTACTGTTTGAAACCAAAGCAGTTTTGATGACACCAACCTTAC
CCATGTGCAAGGAAACATTGGTTTTTCCTTATTACTTTGAAATGAGCAGACATACAGCAT
ACTGAAGATTAGTAAAACTGAAACTAAAAATAAAGTTACAGCACCTTACGAAGTATTTCT
CACATGTAAATTTCATCAAACTAAGGTAGTTTGGCACACACCTATTACATATATGAAGAT
TTTATGGTAACAAAATCATGCACCCGACTTTAAAATTCATATTTGCTGAGCCAATTTTCTA
TAACACCAAAAACACTCATATCTCTCACACACAAATTAACTAATGCCCAGTAACTAAATT
GAAGCTTTCCAGTACTTGCTGCAGATTACTGGAGGTTAGGCAAGACTTTTCCCAGCTAA
TGACACGCAATGGTTGCCATGCAGGAATGAGGGCAGCAGATTCTGAAACCAAAGACAG
GCTGTTTAAAAAATGTACACAAATGGATGACATGTGTGCACAAAAATATCATTCAAATAC
ACACATACAATGATTCTCTCAAATAAATGGTGAACAAAATACGGAGATGGGCCATCACT
CACCAATTTCACAGTTTCTGC

(1093 bp)

ORFinder predicted that contig 498513 sequences consist of 378 bp coding region which started from 56 until 442 bp. The length of predicted protein is 128 amino acids.

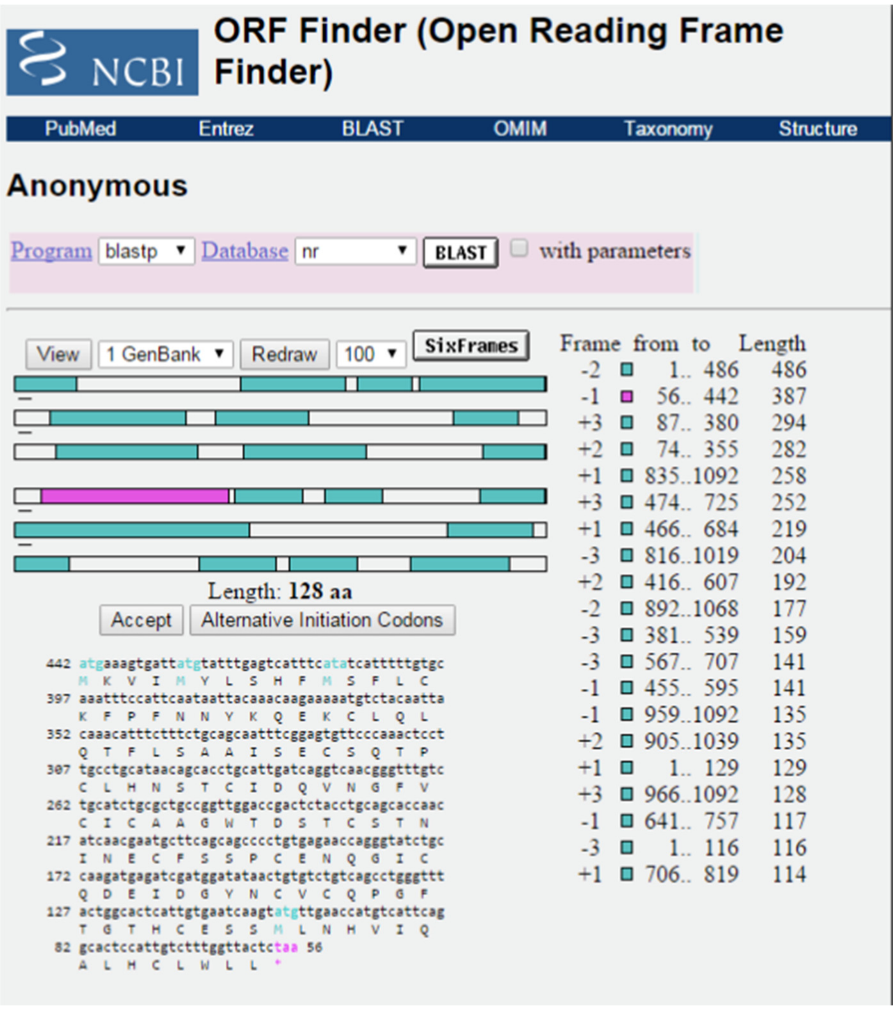

Figure S1.1 Result for predicted coding region according to Contig 498513 sequence. The pink box shown in the diagram is the location of the 387 bp-coding region.

Domain search for this protein showed the presence of calcium-binding EGF-like superfamily in two different locations; domain A from 38 to 72 amino acids and domain B from 75 to 111 amino acids sequences. Calcium ion binding sites (Asn75, Glu78 and Asp92) are located in Domain B (Figure 1.2).

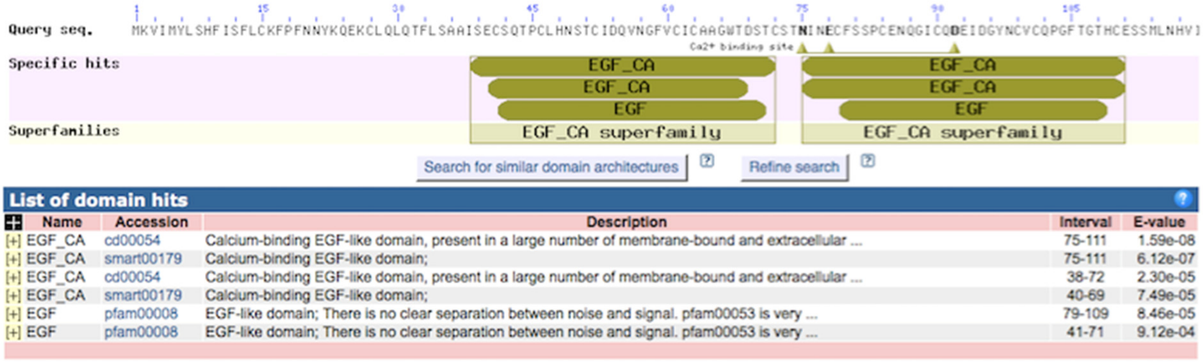

Name	Accession	Description	Interval	E-value
[+] EGF_CA	cd00054	Calcium-binding EGF-like domain, present in a large number of membrane-bound and extracellular ...	75-111	1.59e-08
[+] EGF_CA	smart00179	Calcium-binding EGF-like domain;	75-111	6.12e-07
[+] EGF_CA	cd00054	Calcium-binding EGF-like domain, present in a large number of membrane-bound and extracellular ...	38-72	2.30e-05
[+] EGF_CA	smart00179	Calcium-binding EGF-like domain;	40-69	7.49e-05
[+] EGF	pfam00008	EGF-like domain; There is no clear separation between noise and signal. pfam00053 is very ...	79-109	8.46e-05
[+] EGF	pfam00008	EGF-like domain; There is no clear separation between noise and signal. pfam00053 is very ...	41-71	9.12e-04

Figure S1.2 BLAST result for the domain search hits

Sequence alignment search of the predicted protein showed 57% identity with EGF of *Strongylocentrotus purpuratus* (purple sea urchin).

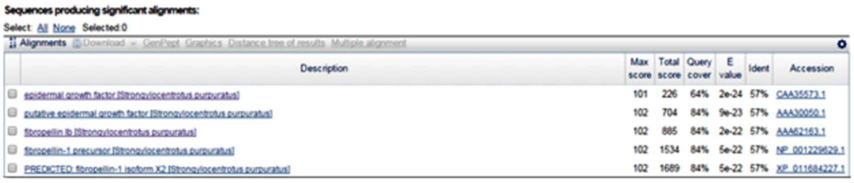

Description	Max score	Total score	Query cover	E value	Ident	Accession
epidermal growth factor [Strongylocentrotus purpuratus]	101	226	64%	2e-24	57%	CAA35573.1
putative epidermal growth factor [Strongylocentrotus purpuratus]	102	704	84%	9e-23	57%	AAA30050.1
fibropellin Ib [Strongylocentrotus purpuratus]	102	885	84%	2e-22	57%	AAA62163.1
fibropellin-1 precursor [Strongylocentrotus purpuratus]	102	1534	84%	5e-22	57%	NP_001229629.1
PREDICTED: fibropellin-1 isoform X2 [Strongylocentrotus purpuratus]	102	1689	84%	5e-22	57%	XP_011684227.1

Figure S1.3 BLAST result for sequence alignments of the predicted protein

B) Protein Modeling of *S. horrens* Protein

The 128-amino acids length of protein sequence which was previously obtained from ORFinder was submitted to I-TASSER webserver for tertiary structure prediction.

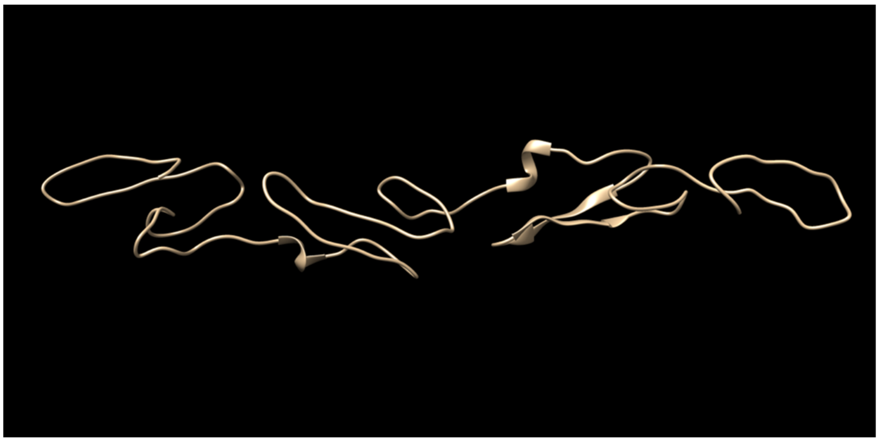

Figure S1.4 Tertiery structure of S. horrens protein modeled by I-TASSER

Structure refinement was made by MoRefiner where the structure was analyzed for hydrogen bonds, backbone protein topology and side chains. Figure 1.5 showed the beta sheets seen at four different regions; Met5, Tyr6, Leu7, Ser8 (coloured in purple), Ser12, Phe13, Leu14, Cys15 (orange), Ser50, Thr51, Cys52, Ile53, Asp54, Gln55 (green) and Gly58, Phe59, Val60, Cys61, Ile62 and Cys63 (yellow).

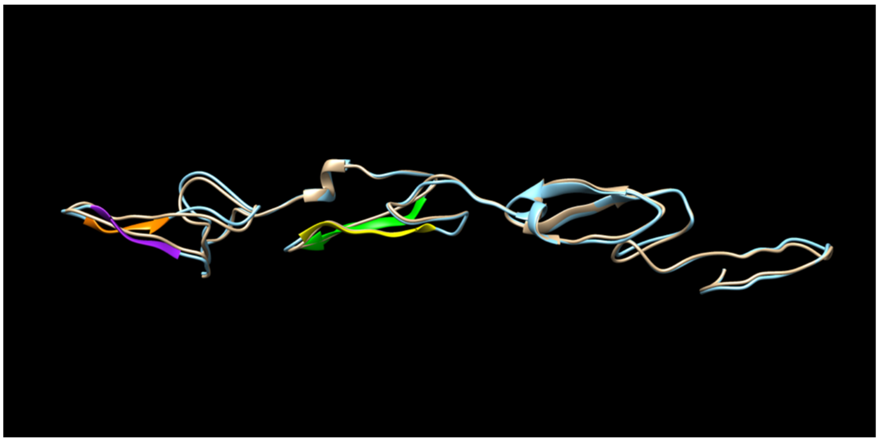

Figure S1.5 Comparison of tertiary structure before undergo structure refinement (gold) and after refinement (blue). The beta sheets coloured in purple, orange, green and yellow are the structures predicted by ModRefiner.

The refined structure was being analyzed for structure validation using MolProbity. The resulting structure was then sent for further analysis in PDBSum webserver where this includes programs like PROCHECK and PROMOTIF. PROCHECK analyzed structure in more details while PROMOTIF generate the structure analysis summary. PROMOTIF identified that *S. horrens* protein contains 6 strands (A, B, C, D, E and F), 6 beta hairpins, 1 helix, 24 beta turns, 7 gamma turns and 3 disulphides.

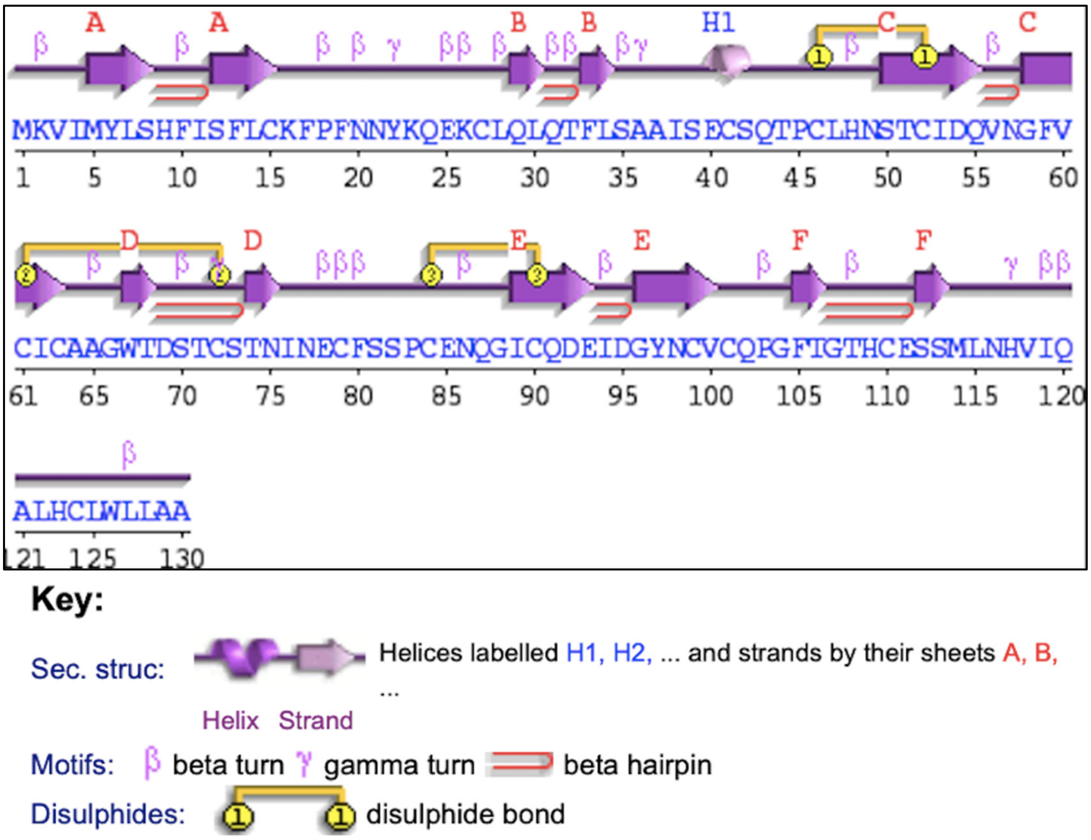

Figure S1.6 Summary of the *S. horrens* protein structure predicted by PROMOTIF

PROCHECK

Ramachandran Plot

w024

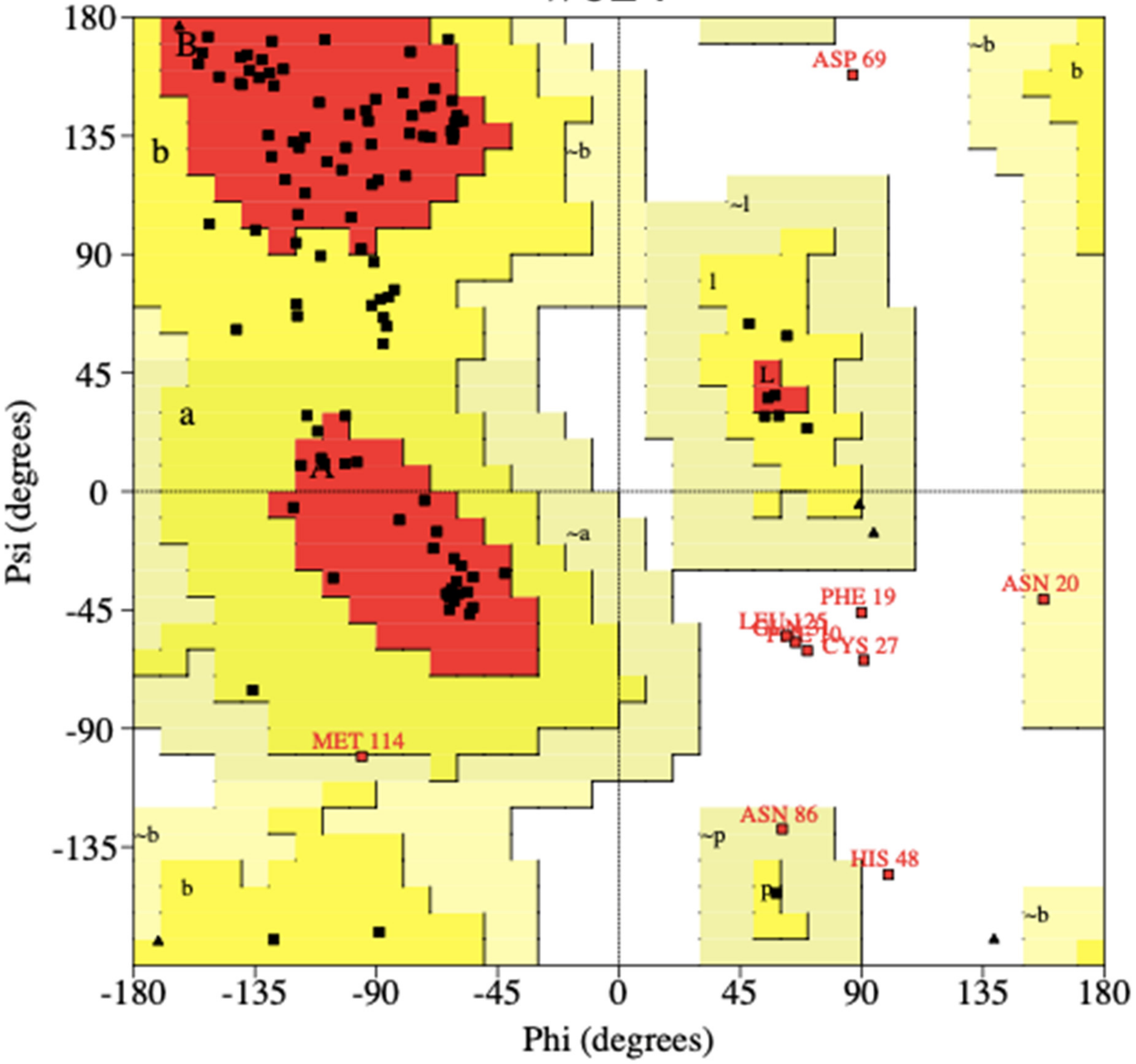

Plot statistics

Residues in most favoured regions [A,B,L]	82	69.5%
Residues in additional allowed regions [a,b,l,p]	26	22.0%
Residues in generously allowed regions [~a,~b,~l,~p]	3	2.5%
Residues in disallowed regions	7	5.9%
	----	------
Number of non-glycine and non-proline residues	118	100.0%
Number of end-residues (excl. Gly and Pro)	2	
Number of glycine residues (shown as triangles)	6	
Number of proline residues	4	

Total number of residues	130	

Based on an analysis of 118 structures of resolution of at least 2.0 Angstroms and R-factor no greater than 20%, a good quality model would be expected to have over 90% in the most favoured regions.

Figure S1.7 Ramachandran plot of *S. horrens* protein structure obtained after PROCHECK analysis

C) Structure comparison with human EGF

Human EGF structure chain C was derived from 1IVO crystal structure. The structure was then aligned with *S. horrens* protein using PyMOL.

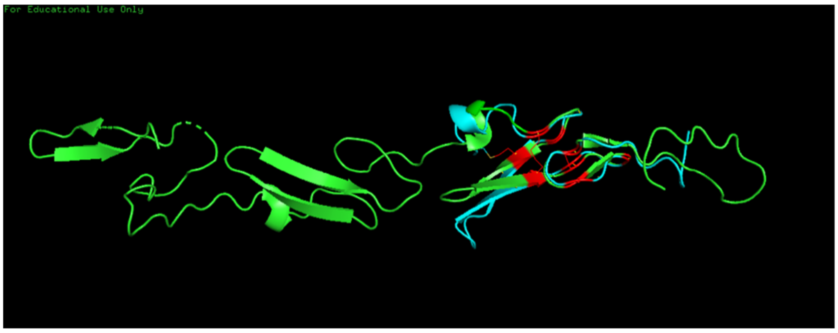

Figure S1.8 Structure alignment of *S. horrens* protein (green) and human EGF 1IVO chain C (blue). The fully conserved residues and three disulphide bonds found in *S. horrens* protein are shown in red.

Sequence alignment of *S. horrens* protein and human EGF resulted in 30 residues of human EGF were aligned from Cys84 to Glu111, which falls within the calcium-binding EGF-like domain B.

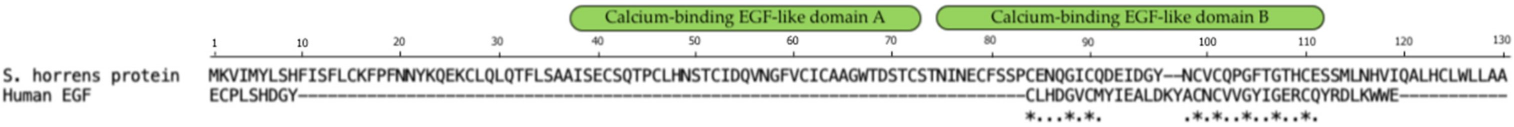

Figure S1.9 Sequence alignment of *S. horrens* protein and human EGF. The asterisk "*" refers to the conserved residues and the dot "." refers to the partially conserved residues.

Based on these structure and sequence alignments, the sequence begins from the calcium-binding EGF-like domain B (Asn 75) until the end of residues aligned with human EGF (Ile119) were named as Sh-EGFl-1 peptide and used for further analysis like binding interaction study through molecular docking. The sequence was NINEC FSSPC ENQGI CQDEI DGYNC VCQPG FTGTH CESSM LNHVI with 45 amino acids length. Sequence homology search using Blastp version 2.7.1 of Sh-EGFl-1 peptide resulted in 31% identity with human EGF structure with E-value of 2 x 10^5 and 73% coverage.

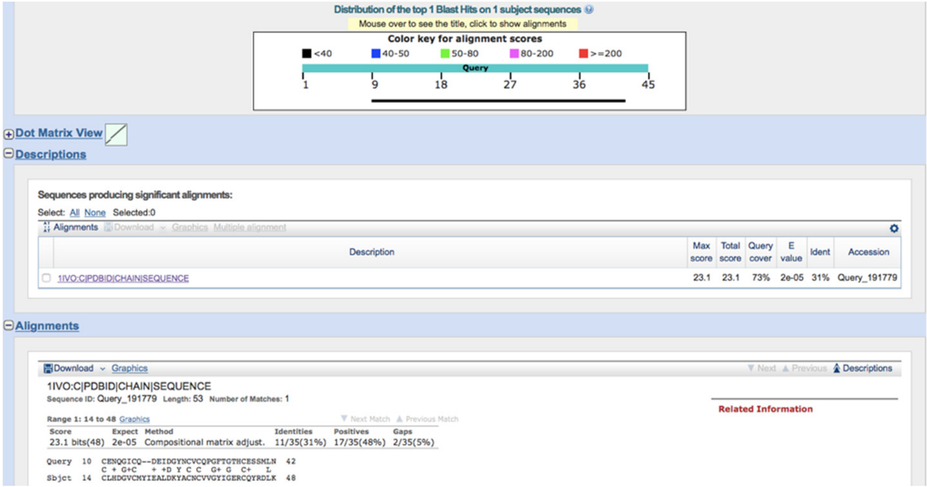

Figure S1.10 Result for sequence homology search using Blastp version 2.7.1 of Sh-EGFI-1 peptide
