# Supplementary material for: Potential of Epidermal Growth Factor-like Peptide from the Sea Cucumber Stichopus horrens to Increase the Growth of Human Cells: In Silico Molecular Docking Approach
Source: Mar Drugs. 2022 Sep 23;20(10):596. doi: 10.3390/md20100596 (PMC9605497; doi:10.3390/md20100596)
Supplement: Supplementary file 1 [file marinedrugs-20-00596-s001.zip › Supplementary Materials S2 - S. horrens protein docking.pdf]

Supplementary Materials S2

Molecular docking of *S. horrens* protein

Before performing docking simulation using HADDOCK, the possible active sites of both molecules (*S. horrens* protein and human EGFR) need to be identified beforehand. The active residues of *S. horrens* protein were predicted using CPORT webserver.

Status: FINISHED

Your prediction was successful

The following predictors finished successfully
PIER (Kufareva et al., 2007)
cons-PPISP (Chen and Zhou, 2005)
PINUP (Liang et al., 2006)
ProMate (Neuvirth et al., 2004)

Predictors were integrated by CPORT:
S.J. de Vries and A.M.J.J. Bonvin
"CPORT: a Consensus Interface Predictor and its Performance in Prediction-driven Docking with HADDOCK."
PlosONE, 6 e17695 (2011)

Please cite all of these papers in your work

Failed predictors
WHISCY, SPPIDER

Predicted residues (active residues in HADDOCK):
6, 8, 13, 14, 77, 79, 80, 81, 82, 83, 84, 90, 99, 106, 114,
125, 126, 127, 128, 129, 130,

Surrounding residues (passive residues in HADDOCK):
5, 7, 9, 10, 11, 12, 15, 16, 25, 26, 30, 75, 76, 78, 85,
86, 87, 88, 89, 91, 92, 93, 96, 97, 98, 100, 101, 102, 104, 105,
107, 108, 109, 111, 112, 115, 116, 117, 118, 122, 123, 124,

Thank you for using CPORT

Download a PDB containing your prediction
Predicted (red) and surrounding (green) residues are in the B-factor (temperature) column

Figure S2.1 Active and passive residues of *S. horrens* protein predicted by CPORT webserver

Mapping of active residues on *S. horrens* protein sequence showed that 48% (10 of 21 residues) were listed in the calcium-binding-like domain B.

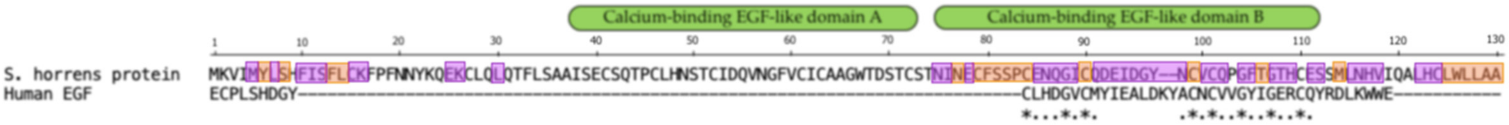

Figure S2.2 Active and passive residues given by CPORT mapped on the sequence of *S. horrens* protein. Active residues were highlighted in orange and passive residues were in purple.

Meanwhile, the active residues of human EGFR 1IVO retrieved from Ogiso et al. (2002) and were employed during docking simulations are: 14, 16, 17, 18, 31, 32, 33, 45, 69, 90, 98, 101, 336, 350, 355, 357, 382, 384, 412, 438.

Molecular docking of *S. horrens* protein and human EGFR was performed using HADDOCK 2.2 webserver. Active residues were given before submitted for docking. In the meantime, the human EGF-EGFR of 1IVO structure were submitted for HADDOCK for validation as well as to get the prediction of binding affinity and dissociation constant (K_d). The active residues for human EGF chain C were retrieved from Ogiso et al. (2002) and were employed during docking simulations are: 6, 7, 8, 9, 10, 11, 12, 13, 14, 15, 16, 17, 18, 19, 20, 21, 22, 23, 24, 25, 26, 27, 28, 29, 30, 31, 32, 33, 41, 43, 45, 47.

The comparison of both docking structures i.e., (1) *S. horrens* protein and human EGFR and (2) human EGF and EGFR were compared in terms of:

a) Interacting residues

The interacting residues were inspected by visualizing the model using PyMOL. Firstly, the binding site was inspected. *S. horrens* protein binds to the right which is domain I and III of EGFR. Then, the active residues were highlighted and inspected to see their distributions in the model.

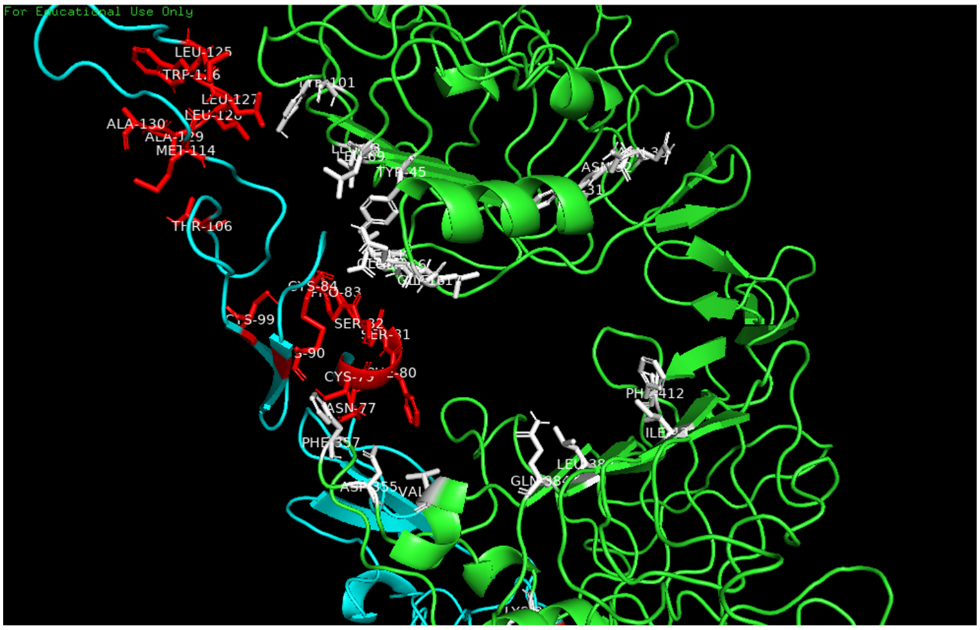

Figure S2.3 Docking model of *S. horrens* protein (blue) with human EGFR (green). The active residues of *S. horrens* protein were shown in red and the active residues of human EGFR (green) were in white.

The binding site of the docking model are found within domain I and III of EGFR. For domain I EGFR, Lys105 formed two hydrogen bonds with Leu127 and Trp126 and in domain III EGFR, Phe357 formed hydrogen bond with Asn77 and at the same time formed hydrophobic interaction with Cys79 and Cys90 of *S. horrens* protein.

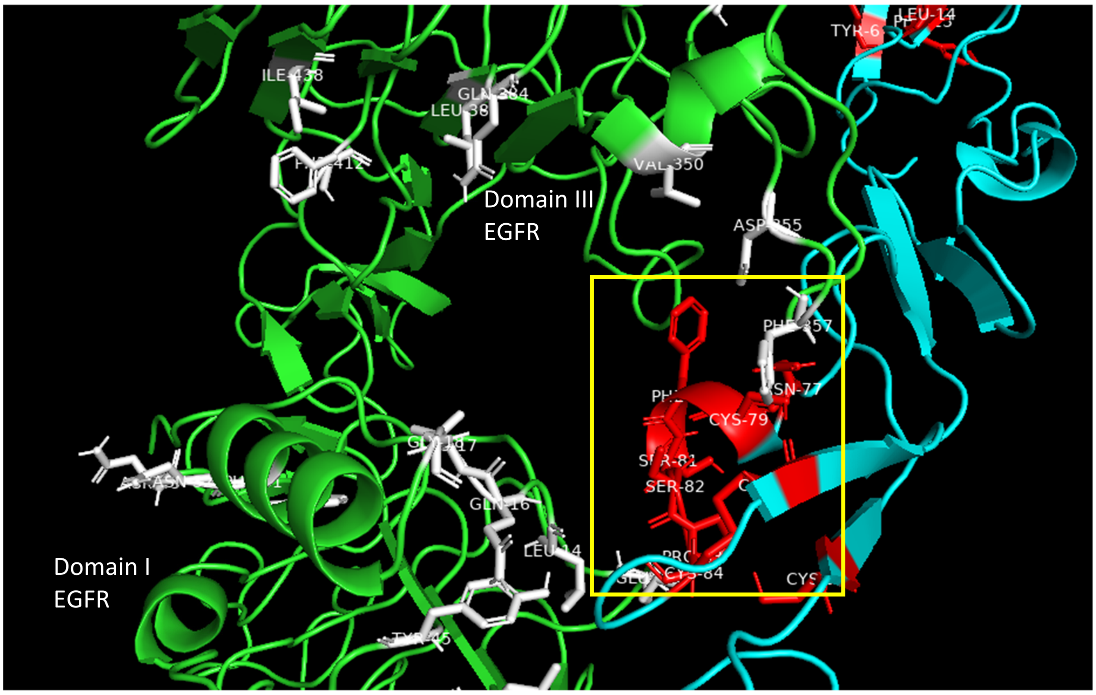

Figure S2.4 The docking model which showed Phe357 on domain III EGFR formed hydrogen bond with Asn77 and at the same time formed hydrophobic interaction with Cys79 and Cys90 of *S. horrens* protein.

b) Number and types of bonding

LigPlot+ version 2.1 program was employed to view interactions in two-dimensional view.

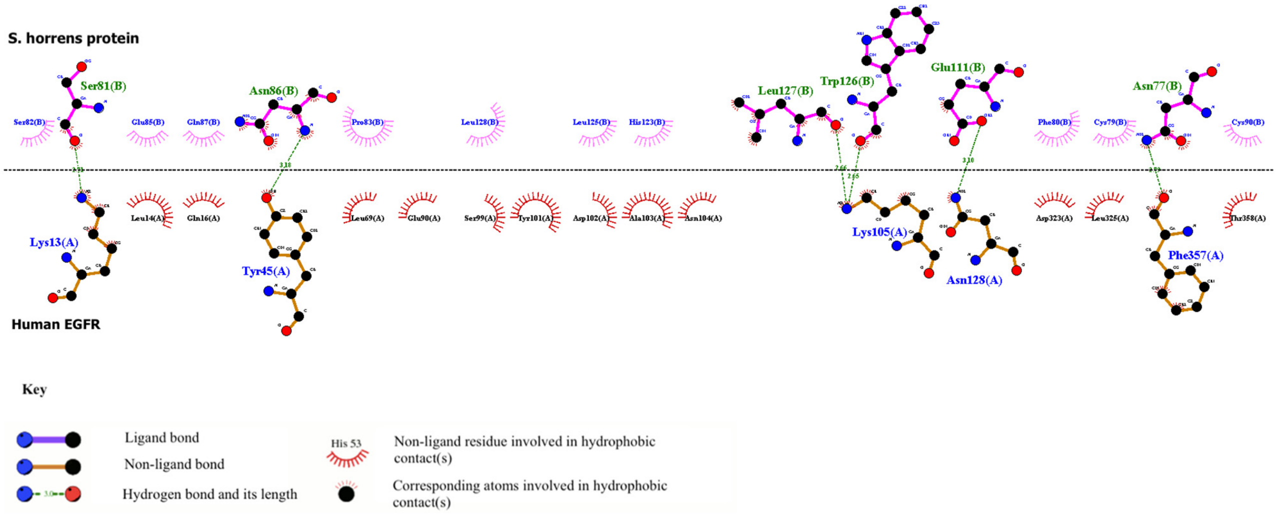

Figure S2.5 Analysis of hydrogen bonds and hydrophobic interactions of *S. horrens* protein and human EGFR in two-dimensional view using LigPlot+

The result from LigPlot+ was presented in tabulated form as below;

Table S2.1 The list of *S. horrens* protein's residues and its type of interactions with human EGFR

Model : *S. horrens* protein and human EGFR					
Interacting residues (among the active residues) with human EGFR					
**Hydrophobic interactions**			**Hydrogen bonds**		
Ser82	-	Lys13	Ser81	-	Lys13
Pro83	-	Glu90, Tyr45	Leu127	-	Lys105
Leu128	-	Tyr101	Trp126	-	Lys105
Leu125	-	Ala103	Asn77	-	Phe357
Phe80	-	Asp323, Leu325			
Cys79	-	Phe357			
Cys90	-	Phe357			
Interacting residues (other than the active residues' list given) with human EGFR					
Glu85	-	Leu14	Asn86	-	Tyr45
Gln87	-	Gln16, Leu14	Glu111	-	Asn128
His123	-	Asp102 Ala103, Asn104,			
Asp86	-	Leu69			

Left column = residues in *S. horrens* protein

Right column = residues in human EGFR

c) RMSD, binding affinity and K_d values

Table S2.2 Comparison of the top three models (Cluster 3, 9,1) generated by HADDOCK with human EGF-EGFR 1IVO

	1IVO	*S. horrens* protein- human EGFR		
		Cluster 3	Cluster 9	Cluster 1
Binding affinity (kcal/mol)	-15.4	-11.2	-14.8	-10.5
Dissociation constant, K_d (M)	5.2×10^{-12}	6.6×10^{-9}	1.5×10^{-11}	1.9×10^{-8}
Number of interacting residues	25	16	18	14
Number of hydrogen bonding	12	6	4	6
Number of residues overlapping with EGF 1IVO	-	3	8	7
Number of residues overlapping with EGFR 1IVO	-	11	15	12
RMSD value (Å)	-	0.625	0.391	0.438
